# Voyages in Vacation.—XVIII

**Published:** 1889-02-09

**Authors:** 


### Voyages in Vacation.—XVIII.

*(Continued from page 287.)*

### NORWAY AND THE LAND OF THE MIDNIGHT SUN.

We have decided to postpone our description of Gothenburg, the Gotta Canal, and Christiania in order to include some account of the Norwegian fiords and the Land of the Midnight Sun. A correspondent, who has made excursions to these regions in the *Victoria*, has written a descriptive sketch of the pleasant holiday thus spent, which cannot fail to interest many readers. This series of articles will be concluded shortly, when it is proposed to publish them in a book form. Any account of the facilities offered by ship and yacht owners for Voyages in Vacation will be welcomed if sent to the Editor of The Hospital during the next week or ten days. Our Correspondent writes: I desire to describe a sixteen days' trip to Norway in the steam yacht *Victoria*? The *Victoria* was an old friend of mine. I had gone in her for a month's cruise to the Azores, Canary Islands, etc., in May, and was delighted to return to her again, and be welcomed as an old friend by the kindly officers and crew. I need say nothing about the yacht herself, except that I consider her perfect in every way, and should like to spend the rest of my life on board her. I do not pretend to give an exact description of our journey, only to put down a few desultory notes of what struck me as interesting.

We left Tilbury on the 21st July, and had a capital passage across the North Sea. It was a little rough as we neared the Dogger Bank, but only sufficiently so to cause a lively movement among the North Sea trawlers, whose dark red and brown sails and black hulls looked most picturesque against the brilliant blue of the sky and the tossing white-capped waves.

It was beautifully warm on the Sunday, and we all took things quietly that day, glad to rest after the bustle of leaving home, and sufficiently amused by the furtive observation of our fellow-passengers. Of course we all eyed each other askance, like Kilkenny cats, for several hours, as English people proverbially do, but, having decided not to fight, we proceeded very quickly to make friends. There were between sixty and seventy passengers on board, and all seemed bent on making up a pleasant and harmonious party. On the Monday, as we were in quite smooth water, we began our games—quoits, bean bags, darts, etc.—which helped to shake us down into friendliness; and in the evening we reached the entrance to the Hardanger Fiord, and our whole attention was turned to the new country.

Early next morning we arrived at Odde. The scenery all up the fiord was inexpressibly lovely. My first idea of Norway was that it was partially submerged, and would either come up again or go down altogether presently; and I felt inclined, in computing the height of the mountains, to begin to count from the bottom of the sea. It is so obviously only the tops of the mountains that meet our eyes, and on landing the idea is carried out by the vegetation on the seashore, which is similar to that found in Switzerland at the height of 3,000 or 4,000 feet above the sea level. The flowers all over the country are exquisitely beautiful, and grow in the greatest profusion.

We landed at Odde at nine a.m., and took carriages, of which there is a variety—the cariole, to hold one; the stolkjar, for two; and the large comfortable carriage with two horses for four persons. We drove up the valley by the side of a rushing river, the windings of which we followed nearly all the way, among huge boulders and overhanging rocks, winding in and out among cloud-capped peaks, passing from time to time great waterfalls, the height and grandeur of which cannot be described.

It must be confessed that the weather was not good, in fact it rained nearly all the way, but the clouds added greatly to the beauty of the mountains, which were constantly appearing and retiring from view, and taking on new shapes of picturesqueness with the ever-varying misty veils that passed across them. One must be prepared for rain in Norway. The inhabitants are, and regulate their conduct accordingly. I was much struck by the ingenious way in which they dry their hay across hurdles, which keep it up from the damp ground. I wonder why we do not try something of the kind in our treacherous English climate. The houses are mostly built of stone boulders, loosely put together and cemented with clay, or of pine logs, with the crevices between filled in with hay, moss, or fern, as they are in the Swiss châlets. They are roofed with turf, over which the grass and wild flowers spread themselves in sweetest, most lavish, decoration. The wooden huts are not as pretty as they might be—the logs are arranged too evenly, without any eye to the picturesque. We had taken our lunch with us from the ship, and saved much valuable time by lunching *en route* during our drive back to her. We returned on board between four and five, rather wet, but happy, and quite satisfied with our long drive through this magnificent country.

Next morning we started early and walked up to the Buarbrae, a small arm of the great Folgefonde Glacier, which comes down to within about two hours' walk from Odde. There was a beautiful green ice cave in it, with a splendid torrent coming from under, and the walk there was most charming. We left Odde at about one o'clock, and went slowly down the lovely Sör Fiord into the Samlë—all parts of the Hardanger—where we stopped for a few minutes at Eide; then up the dark Mauranger Fiord, which was terribly, almost overwhelmingly, grand in the deep evening shadows. One view at the head of it must be for ever impressed upon our minds. Through a break in the dark purple rocks, rising above the rolling masses of cloud and towering far up into the sky, appeared a majestic glacier, glittering in the light of the setting sun, while the mountains round formed a fitting frame for it; the solemn evening shadows resting on rocks and fiord enhanced the glorious whiteness of the great silent field of ice. Truly there are giants in that land, and one cannot wonder that a nation brought up in such surroundings should people the impenetrable vastness with Jotuns, all-powerful mysterious beings evolved from such immensity.

It seemed sacreligious to go down to dinner after such a sight, but the fiord air is keen, and nature is inexorable in more ways than one, and we passed down the companion-way to take up our every-day life again with a sigh of intense pleasure, and a last lingering look at the beautiful waterfall which plunges down the rocks at the entrance to the fiord. We became quite used to waterfalls, and only one of surpassing grace or extraordinary height could elicit more than a passing greeting after a few days of such travelling. The sunsets among the fiords were very beautiful, though we were not favoured with any grand display, except one night, just as we were passing Hornelen, a gigantic rock, over four thousand feet high and bare to the summit: the Alpen Glühn, shone out after the sun had set, and crowned the great hills around us with flaming crimson and gold.

I do not remember which day we reached Bergen—the days were so long and so full of interest that I lost count of time, and I have kept no notes—but I think it was on the Wednesday after we left home. Bergen is very pretty and quaint, the curious churches and picturesque houses of the old quarter are well worth seeing, and the views from the hills around the town are magnificent; of course we did some shopping, and bought silver and wood carving, furs and walking sticks, as all who visit Norway are in duty bound to do; and we drove out to the queer little Fantoft Kirke, shaped like a Chinese pagoda, which the American consul has had erected in his grounds a few miles from the town. The church was so dark inside that it was more like a well than anything else, but we could see that the pillars were rough pines, and that there were some curious frescoes on the rough walls, one in particular I remember, which showed the recording angel in the act of chastising an impenitent sinner, but owing to the exigencies of the paneling the angel and half his birch were in one picture, while the detached portion of his rod was suspended in mid-air, like a comet, in the next panel. The whole building was of stained brown wood—more curious and interesting than pretty, but worth seeing as a specimen of the old Norwegian Stave Kirke, which I am told means "a church with a porch." On Friday we left Bergen by rail for Vossevangen. It takes four hours and a-half to go a very few miles, but the scenery is all grand, so it does not matter. Besides, everybody seems to have time to spare, and it is evidently the event of the day when the train comes into the little villages we pass, and we always stopped six to ten minutes at each to give us time for coffee and the guard an opportunity to see his friends, who come down to every station to have a talk with him. It is a friendly little railway, and the line is most entertaining: you never know where it will go next, it serpentines about in such an alarming manner. You look out of the window and see the tunnel through which you are to pass well away to the left or right of you, at an apparently impossible angle; and just as you are wondering how the train can ever curl round enough to get in there, you feel a jolting rattle of the loosely-coupled carriages, and find that it is slewing round very comfortably after all. It has a very flexible backbone, or it could never accomplish such gymnastics.

*(To be continued.)*

